# Coping with Wolf-Hirschhorn syndrome: quality of life and psychosocial features of family carers

**DOI:** 10.1186/s13023-020-01476-8

**Published:** 2020-10-19

**Authors:** Sarah Berrocoso, Imanol Amayra, Esther Lázaro, Oscar Martínez, Juan Francisco López-Paz, Maitane García, Manuel Pérez, Mohammad Al-Rashaida, Alicia Aurora Rodríguez, Paula Maria Luna, Paula Pérez-Núñez, Raquel Blanco, Julián Nevado

**Affiliations:** 1 Facultad de Psicología y Educación, Neuro-e-Motion, Investigación sobre aspectos Neuropsicológicos y Psicosociales de las Enfermedades Raras, Universidad de Deusto, Avda. Universidades 24, 48007 Bilbao, Spain; 2 Hospital Universitario Central de Asturias, Neurología Pediátrica, Oviedo, Spain; 3 INGEMM Hospital Universitario La Paz Madrid, Instituto de Genética Médica y Molecular; CIBERER, Madrid, Spain

**Keywords:** Wolf-Hirschhorn syndrome, 4p deletion, Caregivers, Quality of life, Coping, Depression, Social support, Spirituality

## Abstract

**Background:**

Wolf-Hirschhorn Syndrome (WHS) is a rare, congenital disease characterized by a distinctive facial phenotype, seizures, intellectual disability and developmental delay, and pre and postnatal growth requiring lifelong care. The psychosocial status of the family caregivers of children diagnosed with WHS is unknown. This study aims to characterize the sociodemographic and psychosocial profile of WHS caregivers and analyze how these variables impact their quality of life (QoL) and well-being.

**Results:**

The sociodemographic and clinical profile of 22 Spanish caregivers of children with WHS and the characteristics of those affected have been described. Significant relationships were found between sociodemographic and psychosocial characteristics among caregivers. The impact on the parents’ QoL and negative relationship with the symptomatology were assessed. The use of engagement strategies such as problem focused coping was associated with improved psychological QoL and social support.

**Conclusions:**

WHS caregivers share similarities in their profile and needs with caregivers of children with other rare diseases. Pychosocial support groups involving parents caring for children with the same disease could improve caregivers’ well-being and QoL by strengthening their social support network and using positive coping styles.

## Background

Wolf-Hirschhorn Syndrome (WHS; ONIM #194190, ORPHA #280) [1] is a congenital malformation disorder first described in 1961 [2]. It was later recognized in 1965 as the syndrome known today [3, 4]. WHS is a rare genetic condition whose incidence is estimated at 1 in 50,000 births [5] and is predominantly female 2:1 [6]. Some authors suspect that incidence may be higher, about 1 per 20,000 births [7, 8].

This syndrome is caused by a variable size deletion of the distal region of the short arm of chromosome 4 (4p16.3) and is therefore also known as 4p-syndrome [9]. It has a phenotype core characterized by distinctive craniofacial features (Greek warrior helmet-shaped face), pre and postnatal growth and psychomotor development retardation, seizures and intellectual disability. Other non-nuclear clinical manifestations may also coexist such as cardiological, visual, auditory, genitourinary or hypotonia problems, among others [10].

WHS is a contiguous gene deletion syndrome whose clinical severity has, for a long time, been linked to deletion size. However, existing chromosomal duplications, variations in genetic sequencing and other aspects may contribute to phenotypic variation in patients [11]. Even though the prognosis of children with WHS seems to be more promising than it was decades ago, mean life expectancy is unknown [12].

At the neurocognitive level, these children are described as having a profile of intellectual disability and language disorders, with difficulty in the expressive type [13]. However, more current studies indicate that one-third of patients with cognitive delay could be classified as mild or moderate [11, 14], showing strong socialization skills, and whose level of communication may improve with time [15, 16]. Furthermore, the most recent cohorts described, such as the Spanish cohort, apparently perform better than in previous descriptions of the syndrome [17].

Seizures are reported as the major source of concern for parents and professionals of children with WHS, with a high prevalence of around 90% of patients, with their first episode within the first 3 years [10]. It can arise regardless of deletion size, although a study by Zollino et al. [11] estimates its incidence in 96% of cases involving children with deletions equal to or greater than 22 Mb.

All the above-mentioned manifestations accompanying this syndrome, its co-morbidities with other diseases and increased vulnerability to immunological issues may worsen these children’s quality of life (QoL) [18]. To date, there are no studies on WHS that carry out objective analysis of this significant variable, both for the children themselves and their parents [19].

QoL is described as a subjective and multidimensional process [20, 21] and it has become a focus of research in recent decades. It has been included in studies of rare diseases (RDs) [22–27] and chronic diseases [28–32] involving both patients and caregivers. In all these cases the child’s condition negatively affected the parents’s QoL to a greater or lesser extent.

Children’s state of health, the task of caring for them over time, changes in family roles and uncertainty regarding the evolution of their children’s illness also lead to stress and emotional issues for parents, such as burnout, anxiety and depression [33, 34]. For all the above reasons, there are an increasing number of studies on variables affecting the improvement of caregivers’ well-being, such as coping strategies and social support. The way parents cope and adjust to their children’s illness is not only important in order to improve their own physical and emotional well-being but has also been linked to improving the child’s well-being [35, 36].

The focus on parents of children with RDs and their situation was limited until recent years [34]. Increased knowledge and understanding of the impact of living with a RD is needed in order to improve good practice guidelines for the care and support of families and professionals [37].

This study had two primary aims. The first aim was to describe the characteristics of parents and children with WHS in a Spain sample and to explore caregiver’s socioemotional status by assessing their QoL, burden, symptoms, spirituality, coping strategies and social network. The second aim, since there is no background on socioemotional status of caregivers, was to determine its impact and differences if compared to normative reference populations as well as to other clinical samples.

## Methods

### Participants

Study participants were contacted via the Spanish Wolf-Hirschhorn Syndrome Association (AESWH). A total of 22 caregivers (parents) of children and young people with confirmed WHS diagnosis were recruited. Participants were included in the sample if they met the following criteria: 1) had a child under the age of 21, with a professionally confirmed diagnosis of WHS; 2) were a family (mother or father) caregiver of a person with WHS and an “intensive carer”, defined as a person who provides a minimum of 20 h of care per week [38]; 3) lived with the person with WHS or had frequent contact with him/her. Participants were excluded who: a) were not resident in Spain; b) were caregivers under the age of 18; c) had uncompensated sensory deficits preventing the assessment protocol from being administered; and d) were illiterate.

### Instruments

A brief interview for the collection of sociodemographic and clinical information was included. Information on caregivers such as age, gender, marital status, education, employment status, relationship with the patient, daily hours dedicated to care, population type and socioeconomic status was collected. Questions relating to age, gender, time of diagnosis, type of communication, and the children’s deletion size of the short arm of chromosome 4 were also included. After this, the psychosocial and clinical tests were administered.

### Caregivers’ QoL

WHOQOL-BREF [21] - Spanish version [39]. This is the World Health Organization’s QoL short version questionnaire, which consists of 24 items in the Likert 5-point response format and evaluates a total of four domains: physical, psychological, social relationships and the environment. It also includes two additional items reflecting the perception of overall QoL and satisfaction with regard to health. Higher scores in the scale indicate greater QoL.

WHOQOL-SRPB – Spanish Version [40]. This 32-item module evaluates eight facets of spirituality, religiousness and personal beliefs in their relationship to QoL and health: connectedness to a spiritual being or force, meaning of life, awe, wholeness and integration, spiritual strength, inner peace/serenity/harmony, hope and optimism, and faith. The response format is type 5-point Likert where higher scores indicate greater use of that domain. The questionnaire design allows it to be administered to individuals with differing ranges of spiritual, religious and/or personal beliefs.

### Functional and symptomatic assessment of the caregiver

Zarit Burden Interview - ZBI [41] – Spanish Version [42]. This is a self-report instrument that assesses the level of caregiver burden. It consists of 22 items that are assessed using a Likert-type scale, where higher scores indicate greater burnout.

The Symptom Checklist-90-R - SCL-90-R [43] – Spanish Version [44]. This is a self-report checklist for overall psychopathological evaluation. It consists of a total of 90 Likert-type items and contains 9 dimensions: Somatization, Obsessive Symptoms, Interpersonal Sensitivity, Depression, Anxiety, Hostility, Phobic Anxiety, Paranoid Ideation and Psychoticism; in addition to three global indices: Global Severity Index, Positive Symptom Total and Positive Symptom Distress Index. A higher score indicates more severe symptoms.

Coping Strategies Inventory - CSI [45] – Spanish Version [46]. This self-report instrument assesses coping strategies. It consists of a total of 40 Likert-type with higher scores indicating greater use of this coping style. The scale it is divided into a hierarchical structure made up of eight primary subscales: problem solving, cognitive restructuring, social support, emotional expression, problem avoidance, wishful thinking, social withdrawal and self-criticism; four secondary subscales: problem or emotion focused engagement or disengagement; and two tertiary subscales: engagement or disengagement.

Social Network Questionnaire - SNQ [47] – Spanish Version [48]. This is a self-report questionnaire aimed at caregivers of chronic patients that assesses the quality and frequency of social contacts, as well as the subjective perception of support received by the social network. It contains a total of 15 items with four response options divided into four subscales: social contacts, affective support, instrumental support and supportive relationships. Higher scores in the scale indicates greater perception of support.

### Procedure

Rectruitment though Spanish Wolf-Hirschhorn Syndrome Association - AESWH began by contacting the organization via e-mail and a face-to-face meeting with AESWH board members. After explaining the objective and the methodology of the project, the AESWH agreed to participate. Then families received detailed information about the aims of the project by e-mail. People who were interested and who fulfilled the inclusion criteria were connected directly to the research team and then were sent an online form by Google Forms website (between October and November 2017). The assessment protocol was estimated to take 45–70 min, starting with the sociodemographic interview and followed by the online instruments. Contact details were provided in the event of any queries arising during completion of the questionnaire, such as doubts about the online platform access. All participants were informed of the study’s goals and ethical conditions; participation in the study was voluntary and there was no remuneration. The participants were required to sign an informed consent form prior to any test. Ethical Approval and Informed Consent: all procedures perfomed in this study were developed in accordance with the ehical standards and with the 1964 Helsinki declaration and its later amendments. This study also complies with national laws and regulations (Act 41/2002, 14th November) and guarantees the confidentiality of participants and their data in accordance with the Organic Law on the Protection of Personal Data (15/1999, 13th December).

### Study design and data analysis

This was a descriptive cross-sectional and correlational study of caregivers for patients diagnosed with WHS.

Statistical Package for Social Sciences, version 25.0, was used for data analysis. Frequencies, percentages (%), mean (M), standard deviations (SD) and ranges were used to describe the sample. The Cronbach Alpha reliability index calculation was collected for all instruments and the Lilliefors corrected Kolmogorov-Smirnov test was used to check normal sample distribution across the study variables. In the bivariate analysis, Pearson’s correlation coefficient was used between normal distributed and continuous variables. Spearman’s coefficients were used for non-normal distributed and ordinal variables. The correlation coefficient was used to measure the relationship between sociodemographic, psychosocial and clinical variables. Aiming to make an approach to a conceptual model on the mechanisms involved in caregiving processes. The independent t-test was used to determine the existence of statistically significant differences with normative reference populations in two variables of importance to the study, using the SCL-90-R and WHOQOL-BREF instruments. Hedge’s g was used to assess the effect size of these differences. In SCL-90-R, Z-Code sample was used to run difference analysis with the study data. This group is made up of people with issues relating to distress or feelings deriving from day-to-day life issues, but without a diagnosable mental disorder [49]. A code Z sample was selected to draw a comparison with a similar population that shown significant clinical distress, but such as caregivers in other studies, they didn’t meet a mental health problem diagnostic criteria. The Mann Whitney U test was used to compare differences in psychosocial and clinical variables based on caregivers’ employment status. The Kruskal-Wallis test was used for differences based on deletion size. The level of significance adopted was 5%, with a 95% confidence interval (95% CI).

## Results

A total of 22 caregivers participated in the study, whose main characteristics, along with those of the children with WHS, are shown in Tables 1 and 2.

**Table 1:** Sociodemographic characteristics of the caregiver sample

	**M (SD)/n (%)**
**Caregivers**	
Gender	
Female	19 (86.4%)
Male	3 (13.6%)
Age	39.73 (7.19)
Marital status	
Married	19 (86.4%)
Single	1 (4.5%)
Divorced	1 (4.5%)
Widowed	1 (4.5%)
Years of education	14.45 (4.60)
Primary or below	1 (4.55%)
Secondary	10 (45.5%)
University	11 (50%)
^a^ Population	
Urban	3 (13.6%)
Intermediate	4 (18.2%)
Rural	15 (68.2%)
Employment status	
Employed	14 (63.6%)
Unemployed	8 (36.4%)
Gave up work for caregiving	
Completely	7 (31.8%)
Partially	5 (22.7%)
Adjustment to day for caregiving	4 (18.2%)
No	3 (13.6)
Others	3 (13.6)
Socioeconomic status	
Low	1 (4.5%)
Lower-middle	10 (45.5%)
Middle	7 (31.8%)
Higher-middle	3 (13.6%)
High	1 (4.5%)
Primary caregiver	
Yes	17 (77.3%)
No	5 (22.7%)
Hours of care	13.19 (7.61)
Care assistance	
No	11 (50%)
Family member	5 (22.7%)
External caregiver	3 (13.6%)
Benefits	2 (9.1%)
Center	1 (4.5%)

SD, standard deviation. ^a^Number of inhabitants per population: urban (>50,000), intermediate (10,000-50,000), rural (<10,000)

**Table 2:** Sociodemographic and clinical characteristics of the patient sample

	**M (SD)/n (%)**	^**a**^**Range**
**Patients**		
Gender		
Female	11 (55%)	
Male	9 (45%)	
Age (months)	81.85 (56.70)	
Birth order		
Only child	10 (50%)	
Firstborn	4 (20%)	
Second child	4 (20%)	
Third or Subsequent child	2 (10%)	
Deletion size		
Small(< 3 Mb)	4 (30.8%)	
Medium (3-9 Mb)	6 (46.2%)	
Large(> 9 Mb)	3 (23.1%)	
^b^Communication skill		
1	9/17 (52.9%)	1.33–18.42
2	3/17 (17.6%)	1.83–10.25
3	3/17 (17.6%)	3.75–13.33
4	2/17 (11.8%).	11.08–11.15
^c^Walking with no help	9/13 (69.2%)	3.75–18.42
^c^Bowel and bladder control	4/12 (33.3%)	10.25–18.42
^c^Feeding autonomy		
With no help	2/20 (16.6%)	4.08–4.42
With help	8/20 (40%)	1.5–18.42

*SD* standard deviation; ^a^Minimum and maximum age range of patients who have reached this milestone; ^b^Communication skill: 1 (does not use words to communicate and/or uses pictograms to do so), 2 (uses gestures and/or one-word utterances, e.g., when asking for water, points to water or says “water”), 3 (uses 2 or 3-word utterances, e.g., “I want water”), 4 (sentences or speech). ^c^2 years old or younger was not considered for some items, as they had not reached the developmental age to such skills

Table 3 shows the mean scores, scale ranges and standard deviations for the different study instruments.

**Table 3:** Clinical characteristics of the caregivers

**Measures**	**Scale/item range**	**Mean (SD)**
**Subjective burnout (*Zarit*)**	0–88	30.18 (10.0)
**Coping strategies (*CSI*)**		
Problem solving (1)	0–20	13.68 (5.86)
Cognitive restructuring (2)	0–20	9.41 (6.15)
Social support (3)	0–20	10.73 (6.39)
Emotional expression (4)	0–20	8.86 (6.58)
Problem avoidance (5)	0–20	4.82 (4.10)
Wishful thinking (6)	0–20	10.10 (5.88)
Social withdrawal (7)	0–20	3.63 (3.0)
Self-criticism (8)	0–20	6.09 (6.59)
Problem focused engagement (1,2)	0–40	23.10 (11.20)
Emotion focused engagement (3,4)	0–40	19.59 (11.92)
Problem focused disengagement (5,6)	0–40	14.91 (8.30)
Emotion focused disengagement (7,8)	0–40	9.73 (8.69)
Engagement (1–4)	0–80	42.68 (21.65)
Disengagement (5–8)	0–80	24.64 (15.74)
^a^ **Quality of Life (*WHOQOL-BREF*)**		
Physical domain	0–100	65.58 (15.61)
Psychological domain	0–100	61.17 (16.93)
Social Relationships	0–100	53.03 (17.55)
Environmental domain	0–100	60.31 (11.52)
Individual’s overall perception of quality of life	0–100	48.86 (19.64)
Individual’s overall perception of their health	0–100	60.23 (19.91)
**Spirituality, religiousness and personal beliefs (*SRPB*)**	0–20	9.91 (2.87)
Spiritual connection	0–5	1.80 (1.11)
Meaning and purpose in life	0–5	3.17 (1.09)
Experiences of awe and wonder	0–5	2.74 (0.78)
Wholeness and integration	0–5	2.72 (0.88)
Spiritual Strength	0–5	2.10 (1.10)
Inner peace	0–5	2.64 (0.93)
Hope	0–5	2.95 (0.69)
Faith	0–5	1.69 (1.09)
**Symptom Checklist 90R (*SCL-90R)***		
Somatization	0–4	1.0 (0.69)
Obsessive-compulsive	0–4	0.85 (0.73)
Interpersonal sensitivity	0–4	0.75 (0.71)
Depression	0–4	1.15 (0.63)
Anxiety	0–4	0.65 (0.65)
Hostility	0–4	0.82 (0.67)
Phobic anxiety	0–4	0.27 (0.40)
Paranoid ideation	0–4	0.61 (0.59)
Pyschoticism	0–4	0.39 (0.39)
Global Severity Index	0–4	0.78 (0.49)
Positive Symptom Total	0–90	39.36 (21.25)
Positive Symptom Distress Index	0–4	1.73 (0.43)
**Social Network (*SNQ*)**	1–4	2.96 (0.59)
Social contact	1–4	2.57 (0.52)
Instrumental support	1–4	3.03 (0.90)
Affective support	1–4	2.95 (0.81)

*SD* Standard deviation. ^a^WHOQOL-BREF: we used transformed scores (4–20 to 0–100) to match the WHOQOL-100

### Quality of life

QoL scores on the WHOQOL-BREF subscales were compared with a primary care patient sample [50] using the independent t-test. As shown in Table 4, the primary care sample obtained a higher score in the physical domain, which is statistically significant, showing no differences for the other domains. The WHOQOL-BREF subscales were also compared with published norms for the normative Spanish sample [39]. The mean scores for the physical, psychological, social relationships and environmental domains on the 4–20 range scale were 14.49 (SD =2.50), 13.79 (SD =2.71), 12.48 (SD =2.81) and 13.65 (SD =1.84), respectively. Our study shows that caregivers had lower scores in all domains.

**Table 4:** Comparisons between the caregivers and primary care sample for the WHOQOL-BREF

**WHOQOL-BREF**	**Caregivers**^**a**^		**Primary care Sample**^**b**^		**Mean differences**	**Effect size (g) a vs. b**
	**Mean**	**SD**	**Mean**	**SD**		
	**(*n*** **= 22)**		**(*n*** **= 1241)**			
**Overall QoL**	2.95	0.79	2.9	0.9	0.05	–
**Physical domain**	65.58	15.61	44.8	17.4	a > b^***^	1.20
**Psychological domain**	61.17	16.93	54.2	17.2	7.00	–
**Social relationships**	53.03	17.55	50.0	20.7	4.44	–
**Environment**	60.31	11.52	56.1	14.8	4.21	–

*SD* standard deviation. Higher scores indicate higher QoL. ^***^p < 0.001. Hedge’ *g* are designated as small (0.20), medium (0.50), and large (0.80). ^a^Caregiver sample of this current study ^b^ Primary care sample findings as published by González et al. [50]

### Clinical characteristics

The caregivers’ responses to SCL-90-R were evaluated against the scores obtained from a normative sample of healthy population [51] and a sample categorized as code Z (previously described in methods section) [49]. The independent t-test was used to determine whether there were any statistically significant differences within a broad range of caregivers’ psychological problems and psychopathological symptoms in comparison to the normative and code Z groups. As shown in Table 5, parents show no differences with the code Z group in Positive Symptom Total, Somatization, Interpersonal Sensitivity and Hostility but reported significantly higher scores for the sample from the normative group. However, group Z obtained a higher score, which is statistically significant, than parents in the Global Severity Index,Obsessive-Compulsive, Depression and Psychoticism but, at the same time, the group of parents recorded a higher average than the normative population. Finally, in the case of Anxiety, Phobic Anxiety and Paranoid Ideation, group Z obtained a higher score than the group of caregivers, and the latter displayed no differences in relation to the normative population.

**Table 5:** Comparisons between caregivers, normative and code Z samples for the SCL-90-R

**SCL-90-R**	**Caregivers**^**a**^		**Normative Sample**^**b**^		**Code Z Sample**^**c**^		**Mean differences**	**Effect size (g) a vs.b**	**Effect size (g) a vs.c**
	**Mean**	**SD**	**Mean**	**SD**	**Mean**	**SD**			
	**(*n*** **= 22)**		**(*n*** **= 530)**		**(*n*** **= 188)**				
**GSI**	0.78	0.49	0.51	0.36	1.20	0.69	a > b^**^ a < c^**^	0.74	0.62
**PST**	39.36	21.25	25.30	14.30	38.81	29.03	a > b^***^	0.96	–
**PSDI**	1.73	0.43	1.75	0.48	2.13	0.67	–	–	–
**SOM**	1.00	0.69	0.55	0.55	1.35	0.86	a > b^***^	0.81	–
**OBS**	0.85	0.73	0.60	0.51	1.43	0.86	a > b^*^ a < c^**^	0.48	0.68
**INT**	0.75	0.71	0.45	0.44	1.13	0.88	a > b^*^	0.66	–
**DEP**	1.15	0.63	0.72	0.45	1.70	0.96	a > b^***^ a < c^**^	0.94	0.59
**ANX**	0.65	0.65	0.52	0.49	1.35	0.81	a < c^***^	–	0.88
**HOS**	0.82	0.67	0.45	0.53	1.07	0.95	a > b^*^	0.69	–
**PHO**	0.27	0.40	0.25	0.36	0.60	0.72	a < c^*^	–	0.48
**PAR**	0.61	0.59	0.47	0.50	1.10	0.89	a < c^*^	–	0.57
**PSY**	0.39	0.39	0.21	0.30	0.74	0.70	a > b^**^ a < c^*^	0.59	0.52

*SD* standard deviation. Higher scores indicate worse functioning on a scale of 1–4. ^***^p < 0.001, ^**^p < 0.01, ^*^p < 0.05. Hedge’ *g* is designated as small (0.20), medium (0.50), and large (0.80). *GSI* Global Severity Index, *PST* Positive Symptom Total, *PSDI* Positive Symptom Distress Index, *SOM* Somatization, *OBS* Obsessive-Compulsive, *INT* Interpersonal Sensitivity, *DEP* Depression, *ANX* Anxiety, *HOS* Hostility, *PHO* Phobic Anxiety, *PAR* Paranoid Ideation, *PSY* Psychoticism. ^a^Caregiver sample of this current study ^b^ Normative sample findings as published by González de Rivera et al., [51]. ^c^Z code sample findings as published by Lozano, Ortiz & González [49]

### Associations between sociodemographic,psychosocial and clinical variables

**Fig. 1:**
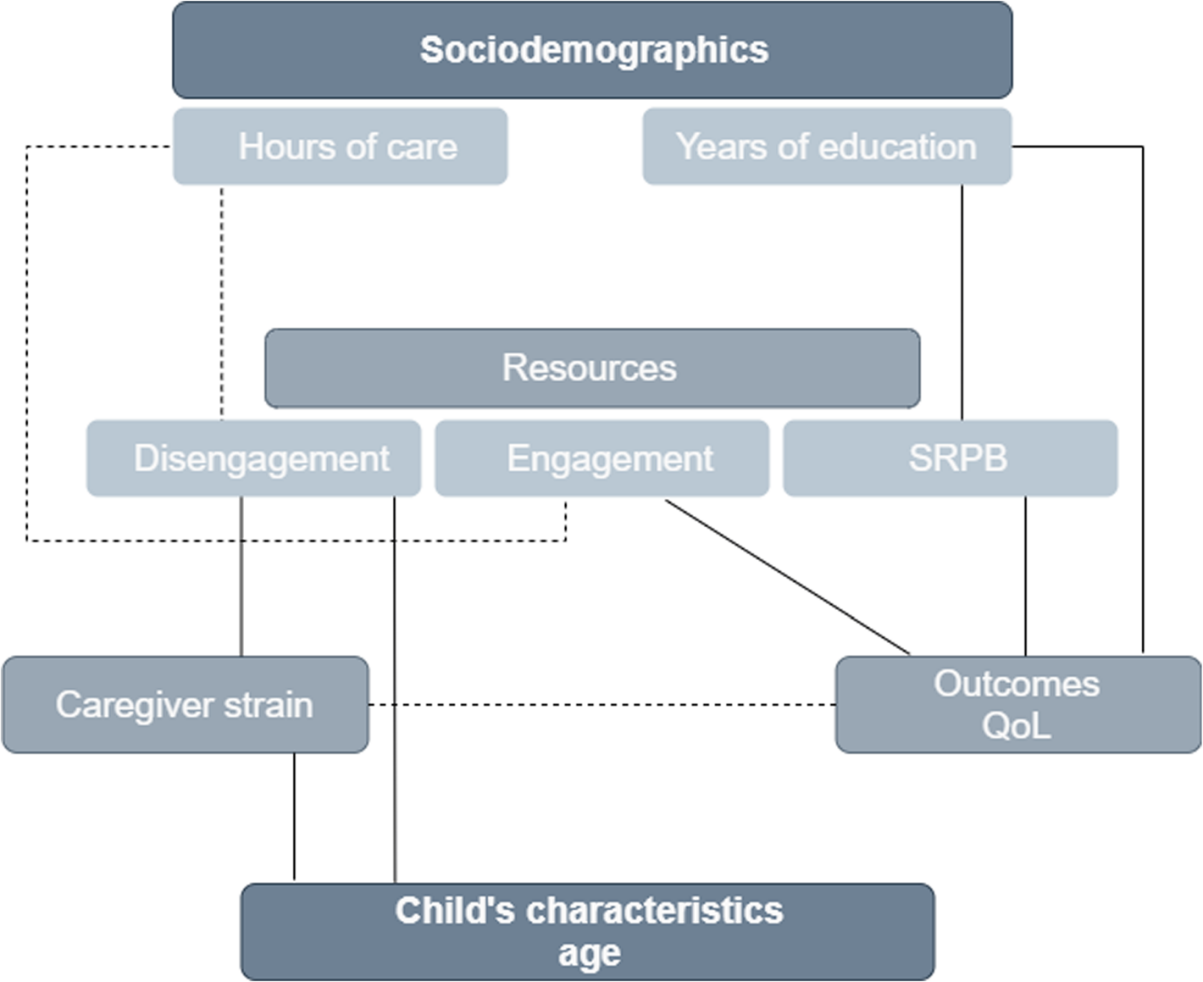
Summarized conceptual model - Mechanisms involved in caregiving processes. Solid lines represent positive correlations and dashed lines negative correlations. SRPB = spirituality, religiousness and personal beliefs

To reach a complete view of psychosocial and clinical status of SWH caregivers’ associations, a conceptual model was created. The five constructs included in the model are: sociodemographic and child characteristics, caregiver strain, resources and outcomes. Each construct includes different variables that could have influence in caregiver status. We have highlighted the significant correlations in the study in a summarized conceptual model in Fig. 1. A complete approach with correlation magnitudes can be seen in Additional files section. Associations of sociodemographics to psychosocial and clinical variables are shown in Additional file 1, for clarity reasons, links between psychosocial and clinical data are shown in Additional file 2. The direction of arrows was chosen based on results and previous published data, but they could include bi-directional effects in a real model that should be tested in future studies.

Differences between sociodemographic characteristics related to psychosocial and clinical data were carried out.

Statistically significant differences were observed in some of the WHOQOL-BREF domain scores and SRPB was based on the caregiver’s town of residence and size of genetic deletion. A Man-Whitney test indicated that the environment domain was significantly greater for caregivers residing in rural areas (Mdn = 65.63) than those in urban or intermediate areas (Mdn = 53.13), (U = 82.5, *p* = 0.032). A Kruskal-Wallis Test was conducted to examine the differences in deletion size. A statistically significant difference (H = 8.34, *p* = 0.015) exists between little and high deletions, (Mdn little = 2.25; Mdn high = 3.50) in wholeness and integration. The same applies to the peace scale (H = 6.49, *p* = 0.39), (Mdn little = 2.13; Mdn high = 3.88).

Finally, significant differences were observed between resources, as coping and social network, based on the caregiver’s town of residence, employment situation and size of deletion. A Man-Whitney test indicated statistically significant differences for caregivers place of residence in problem avoidance (U = 19.5, *p* = 0.017, Mdn rural = 2, Mdn urban = 8), social withdrawal (U = 24.5, *p* = 0.039, Mdn rural = 2, Mdn urban = 6) and problem focused disengagement strategies (U = 23.5, *p* = 0.039, Mdn rural = 14, Mdn urban = 19). Statistically significant differences were also found for problem solving and social support strategies between employed or unemployed caregivers (U = 23.5, *p* = 0.024, Mdn employed = 16, Mdn unemployed = 10) and (U = 26.5, *p* = 0.042, Mdn employed = 15, Mdn unemployed = 7.5). In relation to social support networks, statistically significant relationships were found according to the place of residence for the instrumental and affective support scales (U = 83.0, *p* = 0.032, Mdn rural = 3.67, Mdn urban = 2.33) and (U = 81.5, *p* = 0.039, Mdn rural = 3.4, Mdn urban = 2.4), respectively. A statistically significant difference was also found (H = 6.61, *p* = 0.037) between caregivers’ social contacts and size of small (Mdn = 2.5) and large (Mdn = 3.25) deletion.

## Discussion

### Sociodemographic and psychosocial profile of caregivers

The purpose of analyzing the variables presented in this study was to further our knowledge regarding the sociodemographic and clinical profile of caregivers of children and young people with WHS in Spain. Moreover, to characterize their psychosocial status by analyzing the relationships between quality of life and clinical symptomatology with variables such as burnout, coping strategies and social support networks. Since there is no prior knowledge in this area, the results obtained from this study will be compared with research involving caregivers from related groups, such as chronic illnesses and other RDs.

**Fig. 2:**
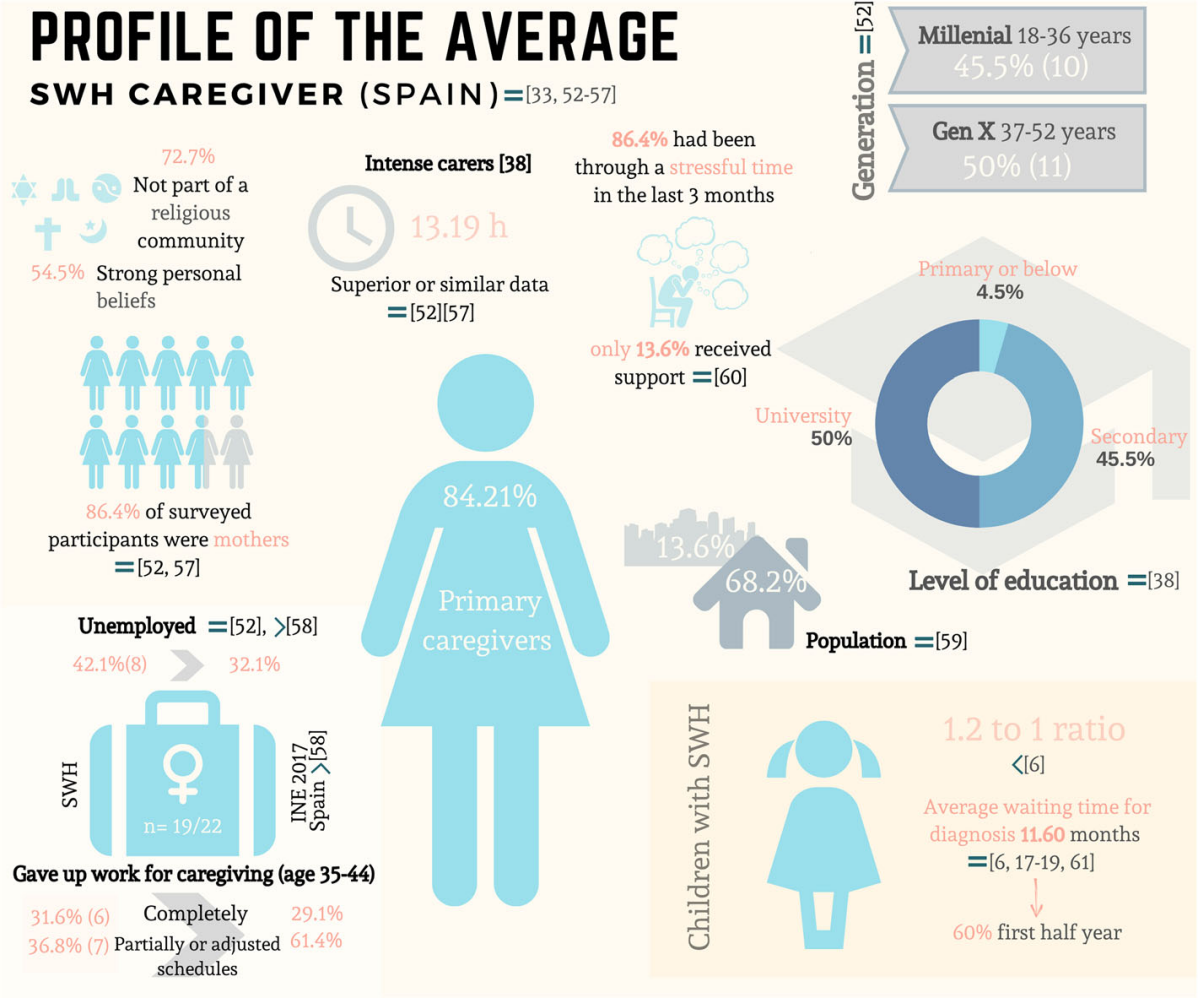
Average profile of WHS caregivers in Spain. This figure shows the data findings of this study and its similarity to other published research: [52–61]. The symbols before number references represent the following interpretation: **a**) > more than in references; **b**) < less than in references; **c**) = comparable to references

Regarding the first aim of the study, Fig. 2 shows a graphical representation that summarizes the average profile of WHS caregivers in Spain and how similar these data are to those shown by other published research.

Also, we have found differences between sociodemographic characteristics related to psychosocial and clinical variables. This type of analysis has become increasingly in recent years. The major findings are related with type of population, hours of care and employment status. First, most of the caregivers in this study lived in a rural area. Previous research highlights that caregivers living in rural areas experience difficulties accessing formal and informal support, such as access to medical services or certain health professionals and/or difficulty contacting social support networks [59]. This geographical constraint has been associated with increased caregiver burnout [54], as well as poorer QoL compared to caregivers living in urban areas. Nonetheless, rural caregivers actively adopt problem focused engagement strategies [62]. No differences were found in this study in the use of problem focused engagement between urban and rural caregivers. However, differences were found in problem focused disengagement strategies such as social withdrawal and problem avoidance. In this case, caregivers living in urban areas obtained higher scores. Caregivers in rural areas perceived more practical and psychological support than caregivers in urban areas, along with a better perception of their environment. Different authors indicate that limited access to support may encourage caregivers to seek out other means to make up for the shortcomings of their environment, such as in our case; greater support in social networks [62, 63].

In the second place, SWH caregivers have shown to spend an average of 13.19 h in caregiving related tasks. More than 6 h of caregiving per day takes up a large part of the caregiver’s “active” day. Such dedication has been associated with a greater risk of physical and psychological distress, as well as depleting socioeconomic resources in the family unit, due for example to changes in employment situation [24, 64]. Indeeddifferent studies with representative samples, have shown that the child’s disease has a clear impact on working life [52]. Most of the Spanish SWH mothers were in a more unfavourable employment situation than their peers as can be observed in Fig. 2. More time spent within the self could improve the increase of internal and external resources. In our study, working caregivers used more problem solving and sought social support, as in other research, where working caregivers made more functional use of coping strategies [65].

### Clinical and developmental profile of children with WHS

The children with WHS in this study presented a level of functional, psychomotor and language skills development similar to previous research on the Spanish cohort [17]. In general, the results obtained in this area are in line with the contributions of authors who point out that motor function and expressive communication in WHS may have a better outcome than the outcome represented in classical studies [66].

With regard to genetic characterization, cases with small or medium-sized deletions were highlighted in this study. Deletion size and/or associated genetic alterations have been linked to the level of development, severity of epilepsy and other phenotypic expressions [10, 11]. This study conducted a preliminary analysis of the relationship between genetic characterization and psychosocial aspects of caregivers. An increased perception of the social network of parents with children with larger deletions was identified; contrary to other studies, where greater symptom severity of children was predictive of less social support perceived by parents [67]. However, when children had larger (mean) deletions, parents reported greater symptomatic distress. Thus, when faced with a larger deletion size, the parents reported more distress but perceived a stronger social support network and used more coping strategies such as problem solving. Moreover, they experienced a greater sense of gratitude, peace and serenity in their lives; these indicators have been seen as a useful resource for coping strategies and improving caregivers’ well-being [68].

### Caregivers’ psychosocial and clinical status

As for the psychosocial and clinical characteristics of WHS caregivers, according to the cutoff points determined by Zarit et al., [41], 95.5% of our caregivers would not be overburdened, as they scored less than or equal to 46 points. However, an average was obtained that is similar to the average of studies involving caregivers of children with other RDs such as Prader Willi and Duchenne Muscular Dystrophy [24, 69, 70]. The ZBI results were analyzed considering the analysis of certain clinically relevant items [71]. Caregivers of children with WHS obtained similar or even higher scores for fear for their relatives’ future 17(77.3%) than other studies of caregivers with children suffering from other RDs, such as Duchenne Muscular Dystrophy [24]. This feeling of uncertainty may be associated with certain WHS characteristics. On the one hand, WSH unknown prognosis [12] and on the other hand, the difficulty in dealing with seizures and their impact could be another factor contributing to this uncertainty, as is the case of caregivers of children suffering from Dravet Syndrome [72]. Sustained uncertainty over time can produce significant emotional distress for the caregiver [71].

In the ZBI responses, 19(86.4%) of caregivers expressed no desire to leave their family member’s care to somebody else. This data is similar to other studies involving infant patients [24] and has been associated as an indicator of parental burnout. Caregivers in these groups may experience feelings of guilt when delegating caregiving to other family members and/or professionals. Some authors have described this as a feeling of omnipotence on the part of the main caregiver, seeing themselves as the most suitable and qualified person to care for their children. Hence, handing this role over to someone else would make them feel guilty [71]. However, other authors have interpreted it as an indicator of parents’ devotion to their caregiving responsibilities despite the high levels of stress and demand involved [24]. A higher burnout score among our caregivers has been associated with a lower perception of social relationships and, in turn, higher levels of symptoms such as Depression, Hostility, Interpersonal Sensitivity and Obsession. We could thus conclude that the caregivers in this study may be experiencing difficulties delegating caregiving to other family members or support persons, which may distance them from their social networks and add to their emotional distress [54, 64, 73, 74].

Since there is no background data on the emotional and clinical aspects experienced by caregivers of children with WHS, we sought to investigate its nature and analyze whether its impact was similar to or greater than the impact in normative reference populations. The most frequent symptom dimensions arising were Depression, Somatization, Obsessive-Compulsive (including unwanted or unavoidable recurrent thoughts or actions), and Hostility (thoughts, feelings, and actions characterizing the negative effects of anger). The results of the caregivers in our study were compared with a sample of healthy population [51] and a code Z population of people with no diagnosis but who were suffering from significant clinical distress [49]. The caregivers obtained a symptom score equivalent to the Z code population in two of the highest scoring variables in the study and scored higher than healthy populations for many of the scale dimensions. The illness’ impact and caregiving situation often leads to psychological distress among caregivers, which often manifests itself in higher levels of depression. These results are in line with numerous studies involving parents of children with other rare or chronic diseases [31, 64, 75–78], and use data obtained in a study involving parents of children with Dravet Syndrome, [60] where, as in our study, despite the distress experienced, caregivers made little use of psychological or psychiatric support (see Fig. 2).

In the study the longer the illness lasted, the greater the increase experienced by parents in symptom dimensions. Several studies have associated children’s age or illness duration with the negative psychosocial adjustment of caregivers [35, 54, 76]. Furthermore, research involving family carers indicates that sleep deprivation, feelings of guilt, loneliness and isolation are closely linked to well-being and QoL [70, 79–83]. The caregivers in this study experienced this range of emotions, which are acknowledged as stressors that impact caregivers and can, at the same time, also affect a child’s QoL.

Understanding the impact of RDs on caregivers’ QoL is important in order to improve socio-healthcare and implement policies for patient and family support and care. In this research, WHS caregivers showed greater deterioration in the QoL environment and social relationships domains. When comparing their scores with the normative reference population, caregivers obtained lower QoL in all domains. However, they showed no differences, with the exception of the physical domain, when compared to a primary care population [50]. On the basis of results from other studies involving parents with chronically ill children, we found similar results were obtained from the parents of children with congenital metabolic disorders [22]. WHS carers presented a lower QoL than other studies of children with epilepsy, attention deficit hyperactivity disorder and cerebral palsy [84, 85]. We have found negative associations between QoL and caregiver’s strain, such us, depression and burnout, nevertheless, resources such as engagement strategies appeared to improve QoL domains. This data can be linked to one of the most recent studies conducted on caregivers of children suffering from RDs [56]. Finally, the parents with higher levels of education seems to displayeda better QoL in some domains. As we said before, associating sociodemographic variables with QoL has become increasingly important, which also reflects the impact of the level of education on the total QoL perceived by the individual [50, 86].

Finally, in terms of coping strategies, emotion-focused coping was the most used in our study and involves the use of direct approach strategies on the stressful event. This coping style is related to improved caregiver status [87]. Moreover, emotion-focused strategies, aimed at relieving the tension created by the stressful event, and which tend to be used when the health condition is perceived as uncontrollable or threatening [88], were the second most widely used by parents in our study. In general, emotion-focused strategies, including disengagement strategies such as social withdrawal and self-criticism, have been associated with an increase in the caregiver’s depressive symptoms, anxiety, burden and distress, as they have in our study [89–91]. Furthermore, emotion-focused disengagement strategies were more visible when the illness lasted longer and, thus, the time devoted to caregiving. This coincides with previous empirical findings [65]. Finally, with regard to coping, religious type has not been evaluated in this study. However, dimensions of spirituality and religiousness linked to QoL that have been shown to be related to some engagement strategies. Hope was seen as a protective factor in situations faced by parents [92]. Indeed, some studies indicate that in the face of uncertainty, parents of children with RDs make greater use of religious coping [34].

These strategies have been effective in seeking social support, positive reinterpretation and growth [93]. In fact, some authors, such as Chivukula [68], suggest that spirituality taken as a multidimensional construct could be a valuable resource for improving caregiver coping and well-being.

### Limitations and future lines

Some of the strengths of our study are that: firstly, to our knowledge, this is the first study focusing on the psychosocial status of parents of children with WHS; secondly, standardized and validated tools have been used, enabling the comparison of our results with other studies, either with normative reference samples or other RDs.

However, the sample size evaluated is small and may not be representative of the entire WHS population. The use of small sample sizes is a limitation often found in the RD population. There is bias toward the use of certain statistical analysis methods, meaning that the interpretation of the significance of results has to be taken with caution and cross-checked with future studies. All the caregivers are members of a National Association for the Syndrome, this is a convenience sample (drawn only from a national patient organization). So that could result in more engaged caregivers, more informed and with access to expert care and consequently could have an impact on caregiver’s coping styles, perceived social support and QoL. These factors may have introduced a bias.

Furthermore, the duration of the assessment protocol was long. It could have been too extensive for some participants to do without fatigue. Even though participants could take short breaks if needed, it is possible that some subjects have lost interest in the full completion of the protocol.

Finally, the use of a cross-sectional design provides us with a still image of a given moment but gives us no information on its evolution throughout the course of time. A longitudinal follow-up study would be of particular interest to this research topic, which, in turn, would increase the sample size so that the results would be more representative of the group of caregivers of children and young people with WHS in Spain.

## Conclusions

The first aim of this study was to describe the characteristics of parents and children with WHS in Spain and to explore the caregivers’ socioemotional status assessing different clinical and psychosocial constructs. The results shows a specific profile of WHS caregivers whose main characteristic coincides with previous literature, for example, gender, age, employment status and level of education. Moreover, the sociodemographic variables included in the study revealed significant relationships with the caregivers’ socioemotional status. The main issues included major time devoted to caregiving tasks and child’s age, which have affected the coping resources of the parents. Whereas years of education and/or living in small areas seems to improve QoL domains and social network.

Regarding the second aim of this study, the caregivers presented a lower QoL than family caregivers with other rare, chronic diseases and normative reference populations. And a higher or equal dimension symptom than normative and primary care populations. Caregivers’ strain has shown to impact caregivers’ resources and outcomes. Considering that the caregiver’s QoL was more favorable toward the use of adaptative coping strategies and disengagement strategies displayed a strong link to caregivers’ psychological distress. We must try to reduce those factors that have shown worsening the caregiver’s QoL.

Having analyzed the situation of caregivers of children with WHS, we have been able to prove that, despite the disease’s particularities and evolution, there are similarities in the characteristics and needs experienced by caregivers in various rare pediatric disorders or related diseases. According to the results of this study, these parents experience emotional distress when coping with the day-to-day care of children with such diseases. This relates to a drop in their perceived QoL. We cannot influence the personal attributes that are already given (child’s age or level of education), but we can intervene on those that can be modified. Cushion the impact of care time, work to reduce symptomatology such as depression, and try to improve resources that promote an adaptive coping with this chronic disease and the care situation.

We propose that interventions, such as psychosocial support groups with other parents, could encourage the improvement of caregivers’ well-being and quality of life by improving their social support network, using positive coping strategies and creating new resources. Further research should consider the use of longitudinal studies and larger samples in order to examine the effect of the associations found in this study.

## Supplementary information

**Additional file 1. **Preliminary conceptual model (part a) – Associations of sociodemographics to psychosocial and clinical data. ^***^p < 0.001, ^**^p < 0.01, ^*^p < 0.05. The italic text represents a Spearman’s correlation coefficients. Solid lines represent positive correlations and dashed lines negative correlations.

**Additional file 2. **Preliminary conceptual model (part b) – Associations between psychosocial and clinical data. ^***^p < 0.001, ^**^p < 0.01, ^*^p < 0.05. The italic text represents a Spearman’s correlation coefficients. Solid lines represent positive correlations, and dashed lines negative correlations. Well known associations between theorical constructs (e.g. SNQ social contacts – QoL social relationships) were omitted from graphical representation.

## Data Availability

The datasets generated and/or analysed during the current study are not publicly available because they belong to the University of Deusto, but are available from the corresponding author (Sarah Berrocoso Cascallana) on reasonable request.

## Abbreviations

SWH: Wolf-Hirschhorn Syndrome
QoL: Quality of Life
RDs: Rare Diseases
