# Supplementary figures and images for: Coping with Wolf-Hirschhorn syndrome: quality of life and psychosocial features of family carers
Source: Orphanet J Rare Dis. 2020 Oct 19;15:293. doi: 10.1186/s13023-020-01476-8 (PMC7574588; doi:10.1186/s13023-020-01476-8)

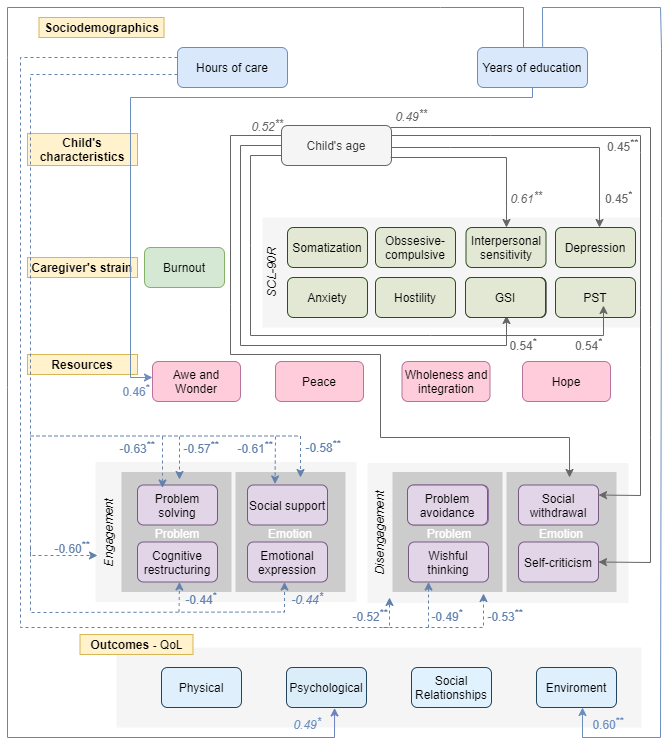

Supplement: Supplementary file 1 — Additional file 1. Preliminary conceptual model (part a) – Associations of sociodemographics to psychosocial and clinical data. ***p < 0.001, **p < 0.01, *p < 0.05. The italic text represents a Spearman’s correlation coefficients. Solid lines represent positive correlations and dashed lines negative correlations. [file 13023_2020_1476_MOESM1_ESM.png]

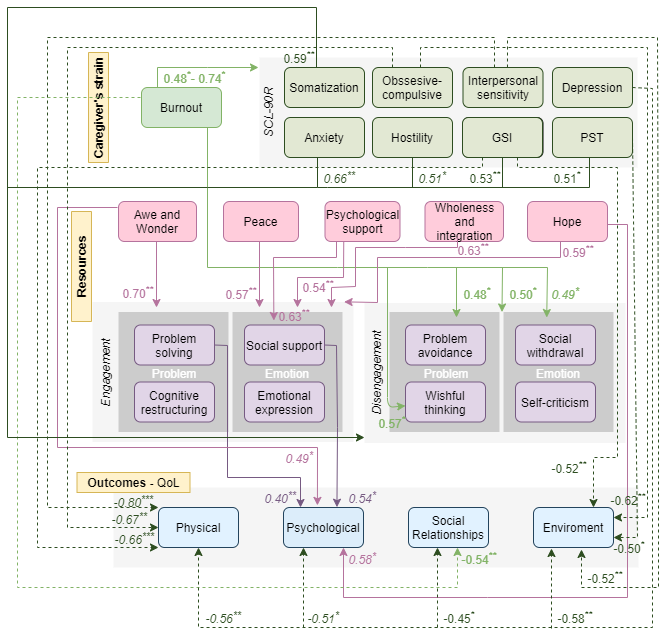

Supplement: Supplementary file 2 — Additional file 2. Preliminary conceptual model (part b) – Associations between psychosocial and clinical data. ***p < 0.001, **p < 0.01, *p < 0.05. The italic text represents a Spearman’s correlation coefficients. Solid lines represent positive correlations, and dashed lines negative correlations. Well known associations between theorical constructs (e.g. SNQ social contacts – QoL social relationships) were omitted from graphical representation. [file 13023_2020_1476_MOESM2_ESM.png]
